# Dual Diagnostic Dilemma: Gitelman Syndrome and Incidental Neuroendocrine Tumor in a Young Adult With Refractory Hypokalemia

**DOI:** 10.7759/cureus.84235

**Published:** 2025-05-16

**Authors:** Abubakar Gapizov, Wajiha Syed, Muhammad Subhan, Ruqiya Bibi, Muhammad Usairam Cheema, Sufyan Mustafa

**Affiliations:** 1 Internal Medicine, Weill Cornell Medicine, New York Presbyterian Brooklyn Methodist Hospital, Brooklyn, USA; 2 Internal Medicine, King Edward Medical University, Lahore, PAK; 3 Medicine, Allama Iqbal Medical College, Lahore, PAK; 4 Internal Medicine, Services Institute of Medical Sciences, Lahore, PAK; 5 Medicine, Dow Medical College, Civil Hospital Karachi, Karachi, PAK

**Keywords:** gitelman syndrome, hypertension and therapy, primitive neuroectodermal tumor (pnet), refractory hypokalemia, type 1 diabetes mellitus

## Abstract

We report the first documented case of Gitelman syndrome coexisting with a metastatic pancreatic neuroendocrine tumor in a 19-year-old male, presenting with severe refractory hypokalemia (K⁺ 1.4-1.7 mmol/L), metabolic alkalosis, and hypomagnesemia. The patient's diagnostic workup revealed inappropriate renal potassium wasting (urinary K⁺ 42 mEq/L), hypocalciuria (urinary Ca²⁺/creatinine ratio <0.1), and elevated fractional chloride excretion (>1%), confirming the diagnosis of Gitelman syndrome. Imaging studies identified a somatostatin receptor-positive Grade 2 pancreatic neuroendocrine tumor (Ki-67 8%) with hepatic metastases, making surgical resection unfeasible. Management comprised high-dose potassium and magnesium supplementation, amiloride, octreotide, and everolimus. On account of disease advancement, initial treatment approaches failed, and peptide receptor radionuclide therapy remained limited for the patient owing to financial constraints. Both Gitelman syndrome and metastatic pancreatic neuroendocrine tumor posed unique challenges that required a coordinated multidisciplinary approach. This case highlights the need for malignancy to be added to the differential diagnosis of persistent electrolyte anomalies. Moreover, it emphasizes the limitations in managing double disease entities in a young individual and the insurmountable hurdles for advanced treatments like peptide receptor radionuclide therapy in underdeveloped nations. This report highlights the importance of further studying the association between the interplay of genetic syndromes (such as Gitelman syndrome) and associated neoplasms, as well as the vital coordination of complex and multidisciplinary management in rare clinical situations.

## Introduction

Hypokalemia is one of the most common electrolyte disorders, occurring in 3%-20% of hospitalized patients, with 60% of hypokalemia cases the result of renal potassium losses [1,2]. Gitelman syndrome (GS) is a rare autosomal recessive disorder due to mutations in the SLC12A3 gene coding for a thiazide-sensitive sodium-chloride cotransporter (NCC) in the distal convoluted tubule [3,4]. This defect impairs the reabsorption of sodium and chloride, leading to compensatory activation of the renin-angiotensin-aldosterone system (RAAS) [3]. As a result, patients develop characteristic electrolyte disturbances, including hypokalemia, metabolic alkalosis, hypomagnesemia, and hypocalciuria [4]. Clinically, GS often presents with muscle cramps, fatigue, or tetany and is typically diagnosed in late childhood or early adulthood [4]. Clinically, the disease can range from mild muscle cramps and fatigue to life-threatening neuromuscular compromise or arrhythmia [4]. Diagnosis is based on biochemical findings - persistent hypokalemia, hypomagnesemia, and low urinary calcium excretion - and is confirmed by genetic testing for SLC12A3 mutations [5]. Management is with potassium and magnesium replacement, sodium and chloride repletion, and avoiding thiazide and loop diuretics that may compound the electrolyte imbalances [6].

Pancreatic neuroendocrine tumors (PNETs) are rare, with an incidence of 0.3/100,000 [7]. Functional PNETs (e.g., insulinomas and VIPomas) can lead to metabolic derangements due to hormone hypersecretion [7]. Non-functioning PNETs comprise close to 30%-50% of cases and present later, with symptoms due to mass effects or metastases rather than hormone imbalance [8]. Functional PNETs are associated with electrolyte imbalances, but rarely, if ever, do non-functional PNETs cause electrolyte imbalances, making significant hypokalemia an unusual finding in such cases [8]. PNETs are diagnosed using imaging modalities, including contrast-enhanced computed tomography (CT) or magnetic resonance imaging (MRI), functional imaging with somatostatin receptor (SSTR)-based scans (e.g., gallium-68-dota-octapeptide positron-emission tomography), and histopathologic confirmation with biopsy [9]. Management involves surgical resection in localized disease and targeted therapy, including somatostatin analogs, mTOR inhibitors, or peptide receptor radionuclide therapy (PRRT) in advanced disease [10]. This unique GS and PNET case report contributes to the literature by highlighting previously unrecognized potential diagnostic and therapeutic barriers associated with dual etiology electrolyte abnormalities. This serves as an important reminder to keep renal tubular disorders on the list of differential diagnoses when evaluating patients for resistant hypokalemia and the need to consider non-functional PNETs as causes of electrolyte disturbance.

## Case presentation

A 19-year-old man, a first-year medical student, was referred for evaluation of progressive fatigue and muscle cramps that had developed over the prior six months and were interfering with his daily activities and academic performance. He also complained of recurrent dizziness that occurred two to three times per week but denied a history of diarrhea, vomiting, diuretic use, or profuse sweating. His medical history was significant for frequent hospital admissions for severe hypokalemia, including serum potassium as low as 1.4 mmol/L. Despite the supplementation of electrolytes, his symptoms did not go away, which led him to a detailed clinical as well as laboratory assessment.

**Figure 1:**
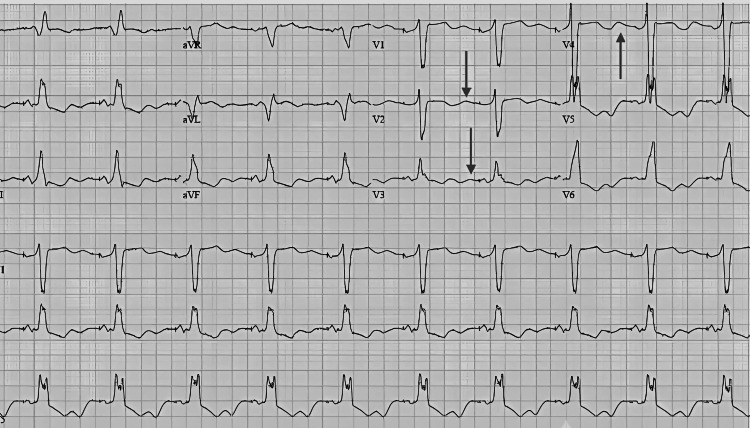
Electrocardiogram changes in a hypokalemic patient The black arrows indicate U waves

On physical examination, his body mass index (BMI) was 21.4 kg/m², and his blood pressure was 108/62 mmHg, consistent with mild volume depletion but not orthostatic hypotension. Neuromuscular examination showed a negative Chvostek’s sign but a positive Trousseau’s sign, suggesting underlying electrolyte abnormalities in the patient that pointed toward hypocalcemia as well as hypomagnesemia. No abdominal masses, organomegaly, or lymphadenopathy were observed. Auscultation of the heart was normal, and there were no signs of hyperpigmentation or flushing, indicating functional neuroendocrine activity. This patient was eventually diagnosed with GS after a stepwise diagnostic workup. The laboratory investigations showed persistent severe hypokalemia and metabolic alkalosis as well as hypomagnesemia, which is routinely seen in GS. Figure 1 shows the electrocardiogram (ECG) with wide QRS, long QT, and prominent U waves.

The patient exhibited inappropriately high renal potassium losses despite profound hypokalemia, as indicated by a urinary potassium excretion of 42 mEq/L and a urinary potassium-to-creatinine ratio exceeding 13 mEq/g-findings that are characteristic of potassium-wasting tubulopathies such as GS. Increased urinary chloride concentration was in line with the diagnosis of saline-unresponsive metabolic alkalosis, a defining feature of GS. Furthermore, the peak FECl⁻ exceeding 1% helped differentiate GS from intrinsic volume depletion states. The patient’s calcium levels were evaluated, with normal serum calcium and severely low urinary calcium-to-creatinine ratio (<0.1), excluding Bartter syndrome and reinforcing the diagnosis of GS. This systematic diagnostic investigation confirmed GS as the etiology of the patient’s electrolyte derangements and excluded other important differentials that may cause similar presentations. Table 1 shows the interpretation of laboratory results during the diagnostic workup of this patient. These values aided in the diagnosis of GS and differentiated GS from other conditions.

**Table 1:** Diagnostic workup and lab findings for Gitelman syndrome (GS)

Laboratory test	Result	Normal range	Interpretation
Serum potassium	Low (1.7 mmol/L)	3.5–5.0 mmol/L	Persistent hypokalemia, a hallmark of GS
Serum bicarbonate	Elevated (34 mmol/L)	22–29 mmol/L	Metabolic alkalosis
Serum magnesium	Low (1.3 mg/dL)	1.7–2.2 mg/dL	Hypomagnesemia, common feature of GS
Urinary potassium excretion	Elevated (42 mEq/L)	< 20 mEq/L (increased excretion in GS)	Inappropriate renal potassium wasting despite low serum potassium
Urinary potassium-to-creatinine ratio	High (> 13 mEq/g)	< 13 mEq/g	Indicative of excessive potassium loss via kidneys
Urinary chloride concentration	Elevated (> 25 mEq/L)	< 25 mEq/L	Saline unresponsive metabolic alkalosis, supporting GS
Fractional chloride excretion (FECl⁻)	Elevated (> 1%)	< 1%	Differentiates GS from volume depletion states
Serum calcium	Normal	8.5–10.5 mg/dL	Helps exclude Bartter syndrome, which is associated with altered calcium levels
Urinary calcium-to-creatinine ratio	Low (< 0.1)	0.1–0.2	Distinguishing feature from Bartter syndrome, consistent with GS

**Figure 2:**
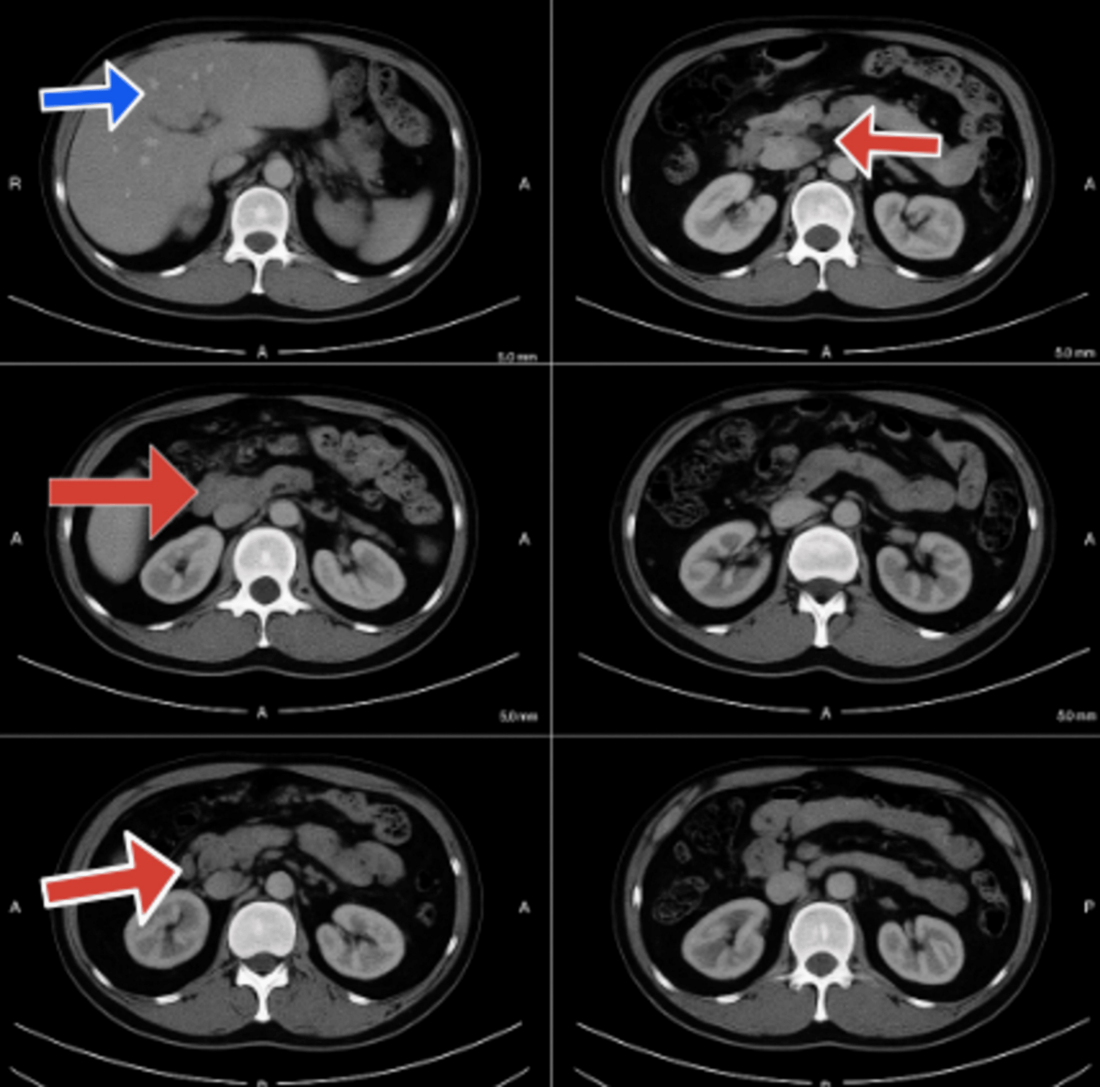
CT abdomen showing pancreatic mass with liver metastasis CT: Computerized Tomography The red arrows show the pancreatic mass, while the blue arrow shows the hepatic metastatic lesions.

Figure 2 shows the contrast-enhanced CT of the abdomen, which shows a heterogeneously enhancing mass lesion, measuring 57 × 48 mm, involving the distal body and tail of the pancreas, with associated pancreatic tail atrophy.

**Figure 3:**
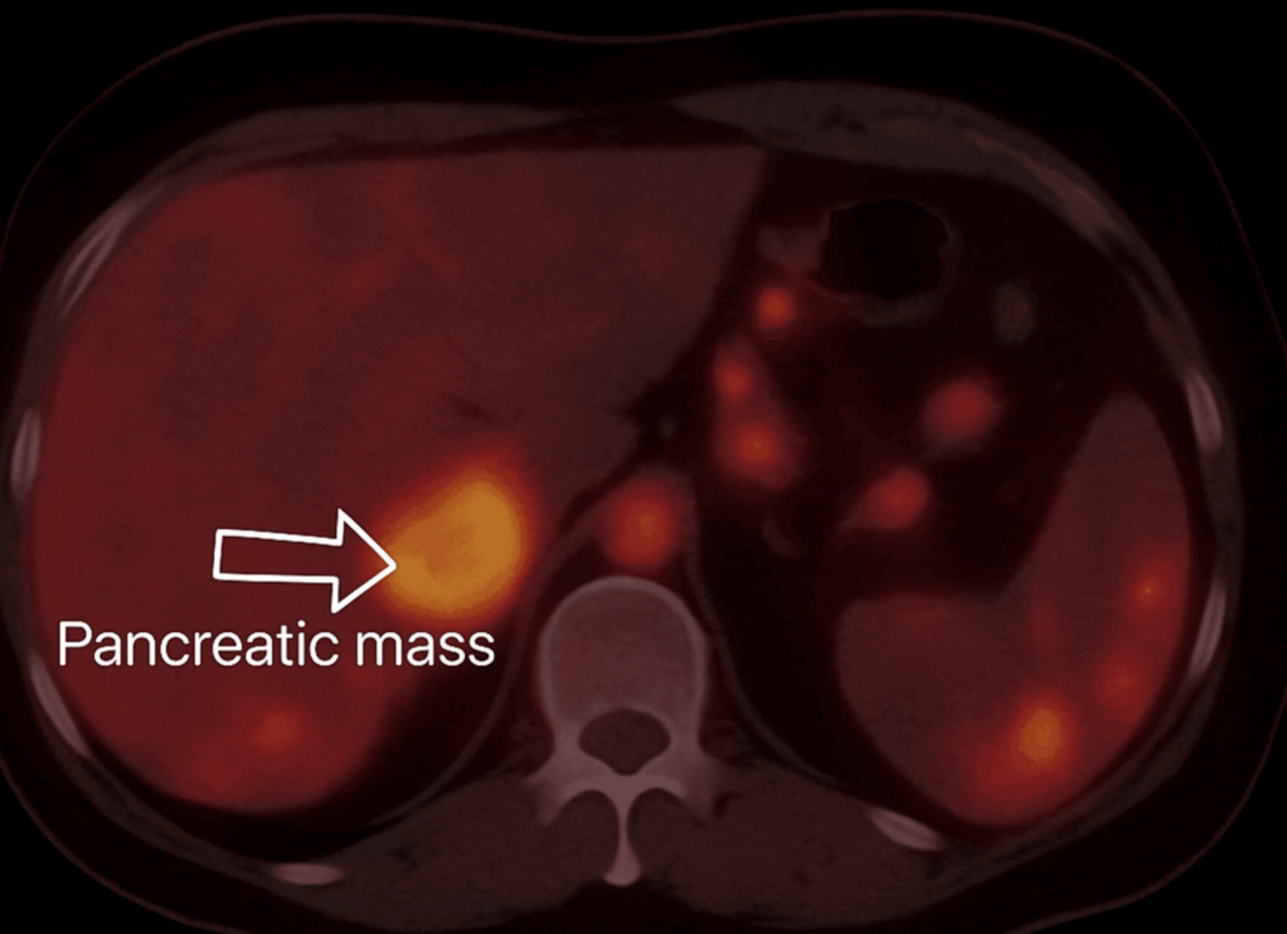
⁶⁸Ga-DOTATATE PET scan showing SSTR-expressing pancreatic and hepatic and splenic lesions consistent with net PET: Positron Emission Tomography, ⁶⁸Ga: Gallium-68 (a radiotracer isotope), DOTATATE: DOTA-[Tyr³]-octreotate (a somatostatin analog used for imaging neuroendocrine tumors), SSTR: Somatostatin Receptor, NET: Neuroendocrine Tumor The arrow shows the pancreatic tumor.

The lesion fully enveloped the splenic artery (>180 degrees), partially enveloped the celiac axis (>90 degrees), and enveloped the superior mesenteric artery (SMA) (90 degrees). The lesion appeared intimately involved with the pylorus and proximal duodenum, with mild haziness of the intervening fat planes concerned with local invasion. The liver showed multiple hyperdense foci with the most significant presence in segment V (21 × 15 mm), consistent with hepatic metastases. No abdominopelvic ascites or lymphadenopathy was noted, nor were there any suspicious bone lesions. Subsequent assessment via ^68^Ga-DOTA-[Tyr³]-octreotate (DOTATATE) positron emission tomography (PET) scan (Figure 3) exhibited intense SSTR expression in both the pancreatic mass and the metastatic liver lesions (Krenning score: 4), consistent with a well-differentiated neuroendocrine tumor (NET).

**Figure 4:**
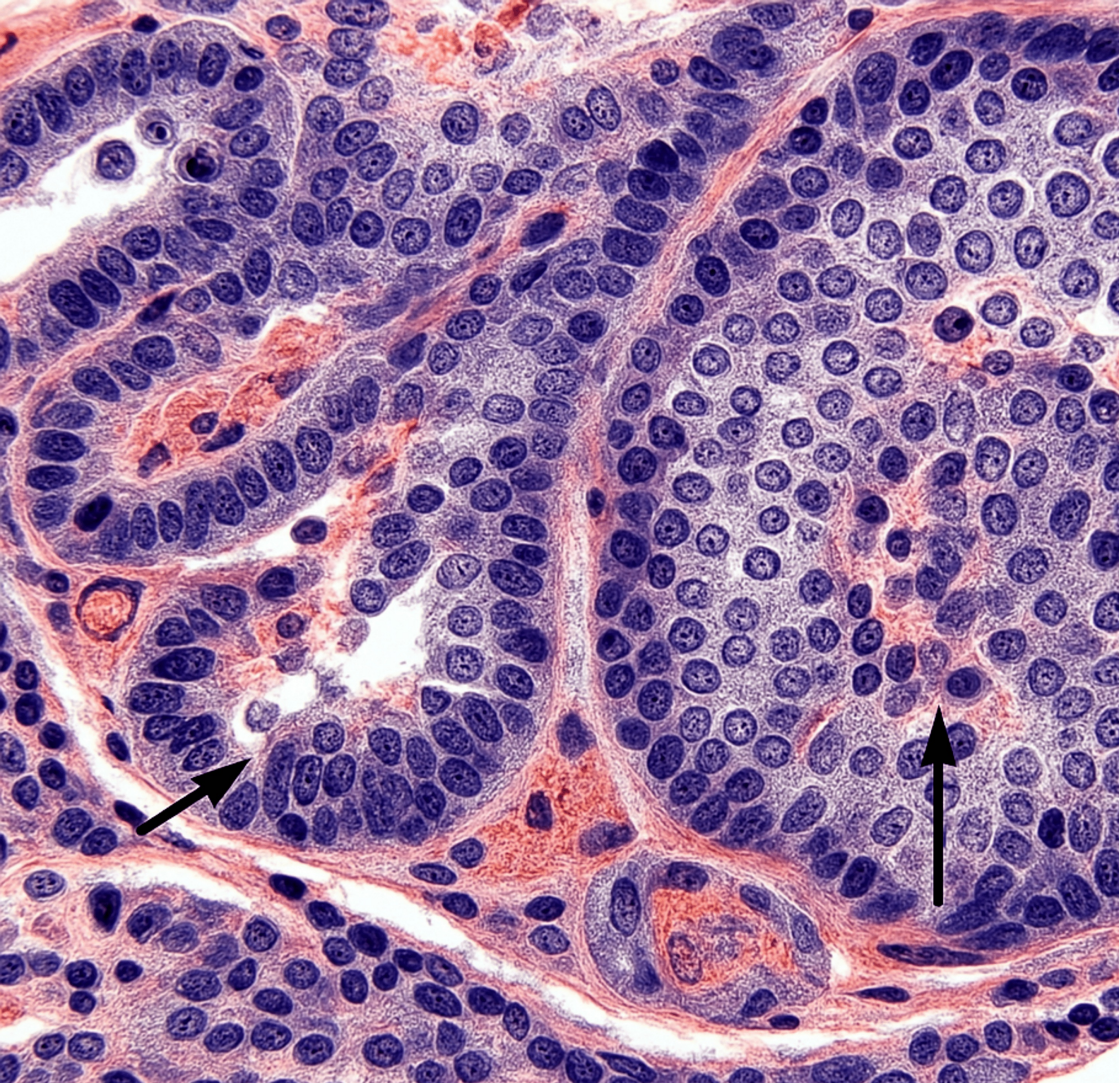
Histopathological image of pancreatic neuroendocrine tumor The black arrows indicate nests of uniform, round-to-oval neuroendocrine cells with salt-and-pepper chromatin and minimal atypia, characteristic features of a well-differentiated pancreatic neuroendocrine tumor (PNET).

A core biopsy of the pancreatic mass under ultrasound guidance revealed a Grade 2 NET (WHO classification 2017) with a mitotic index of 3/10 HPFs and a Ki-67 proliferation index of 8%. The tumor was positive for synaptophysin (+++), CD56 (+), and SSTR2a (++), confirming neuroendocrine differentiation by immunohistochemical staining. Figure 4 shows the hematoxylin and eosin (H&E) stained section of pancreatic tissue at high magnification, demonstrating classic features of PDAC. The image highlights irregular glandular structures lined by pleomorphic epithelial cells with hyperchromatic nuclei set in a desmoplastic stroma.

Per guidelines on the treatment of PNETs, surgical resection was not possible due to the significant vascular encasement and hepatic metastases. Instead, they took a systemic therapy approach: IV potassium chloride (40 mEq/hour) and magnesium sulfate infusions were given with continuous EKG monitoring. Chronic management included high-dose oral potassium chloride (120 mEq/day) and magnesium oxide (600 mg/day) with amiloride (10 mg twice daily) to minimize renal potassium wasting. Somatostatin analog therapy (Octreotide LAR 30 mg monthly) was applied to control tumor progression. Per guidelines for unresectable, progressive PNETs, targeted therapy (everolimus 10 mg daily) was started three months later. In this case, 177Lu-DOTATATE PRRT therapy was considered as evidence of disease progression, given the high SSTR expression. Liver-directed therapies, transcatheter arterial chemoembolization (TACE), were contemplated for enlarged hepatic metastases. Severe electrolyte imbalances improved considerably upon chronic oral supplementation with potassium and magnesium over six weeks. Endocrine therapy alone was given, but disease progression was seen on imaging after six months of follow-up, with investigational options considered. Due to financial constraints, PRRT could not be pursued, limiting further treatment options.

## Discussion

We report the first documented case of GS associated with PNET to date. In our patient, persistent hypokalemia led to an extensive diagnostic workup, resulting in the diagnosis of malignancy in advanced stages. The exact mechanism by which GS and PNET are related is not known; however, it may be attributed to paraneoplastic phenomena or merely a coincidence. This case highlights the need for a comprehensive investigation of persistent electrolyte derangements, as these can be harbingers of an occult malignancy.

GS is an autosomal recessive tubulopathy associated with hypokalemia, metabolic alkalosis, hypomagnesemia, and hypocalciuria, resulting from mutations in the SLC12A3 gene encoding the thiazide-sensitive sodium−chloride cotransporter localized to the distal convoluted tubule [1,2]. Although GS is usually considered a benign condition, severe presentations can lead to significant morbidity due to extreme electrolyte derangements [2]. PNETs are infrequent neuroendocrine neoplasms that originate from the islet tissue of the pancreas with a highly heterogeneous clinical course and can be determined by tumor grade, metastatic load, and SSTR expression [7]. PNETs typically do not produce electrolyte abnormalities, unlike GS [7]. Paraneoplastic syndromes, including those impacting electrolyte homeostasis, have been reported in various malignancies [8]. This supports the potential presence of a paraneoplastic mechanism driving the electrolyte derangements noted in our patient [8]. Although no previous publications have associated GS with PNET, this case highlights the potentially underreported renal and metabolic sequelae of neuroendocrine malignancy.

The results from this case have important clinical implications. First, they underscore that a thorough diagnostic evaluation should be a standard part of the workup in patients with refractory hypokalemia, which may herald an underlying malignant process. Second, this case provides an opportunity to distinguish GS from other causes of renal K+ wasting syndromes. A careful workup, including urinary potassium excretion, evaluation of chloride handling, and calcium metabolism, ultimately led to the correct diagnosis of GS, which was later confirmed by genetic testing. Fluid and electrolyte replacement is aggressive in the management of GS, particularly with potassium and magnesium. The identification of PNET in this case highlights the need for a multidisciplinary approach, including consideration of tumor-directed therapies such as PRRT for SSTR-positive cases. PRRT, however, is generally very costly, and that is also the case in lower-income, resource-limited countries, where access to PRRT is limited by its cost and availability. The specific nature of this case as a single-patient report means that the data presented are difficult to generalize. No data validates the correlation of the association between GS and PNET, and the possible mechanism that links them both remains speculative, requiring additional studies to identify the putative physiopathological pathways. Future studies should investigate whether similar cases also exist and whether there is a more general association between inherited tubulopathies and NETs. We present this case report to highlight previously unreported manifestations of GS and PNET and to emphasize the need for comprehensive metabolic workup in the face of persistent hypokalemia. By recognizing electrolyte disturbances as potential paraneoplastic manifestations, clinicians may achieve earlier diagnosis and improved management of underlying malignancies.

## Conclusions

This case represents the first reported coexistence of GS and a metastatic PNET, highlighting the critical need to evaluate refractory hypokalemia for underlying malignancy. The diagnosis of GS was confirmed through classic biochemical findings (hypokalemia, metabolic alkalosis, hypomagnesemia, and hypocalciuria), while advanced imaging and histopathology identified an aggressive, SSTR-positive PNET. Electrolyte instabilities and unresectable disease complicated the management of the patient, thus requiring a multidisciplinary approach, including nephrology and oncology. This report highlights that persistent electrolyte disturbances, even in keeping with hereditary tubulopathies such as GS, warrant evaluation for secondary causes, including paraneoplastic syndrome. Financial and logistical barriers (e.g., limited PRRT access) can significantly impact outcomes in advanced NETs. Future studies should explore potential mechanistic links between GS and PNETs, particularly whether tumor-derived factors exacerbate renal electrolyte wasting. Clinicians should maintain a high index of suspicion for dual pathologies in similar presentations, ensuring timely intervention for both metabolic and oncologic components.

## References

[1] Chen SY, Jie N (2022). Gitelman syndrome: a case report. World J Clin Cases.

[2] Huang X, Wu M, Mou L, Zhang Y, Jiang J (2023). Gitelman syndrome combined with diabetes mellitus: a case report and literature review. Medicine (Baltimore).

[3] Reyes JV, Medina PM (2022). Renal calcium and magnesium handling in Gitelman syndrome. Am J Transl Res.

[4] Zhang J, Liu F, Tu J (2022). Gitelman syndrome in pregnancy: a case series. J Matern Fetal Neonatal Med.

[5] Pandya S, Shah S, Dalal S (2023). Gitelman syndrome presenting with cerebellar ataxia and tetany. Indian J Nephrol.

[6] Chargui S, Houli R, Ounissi M, Ben Hamida F, Harzallah A, Abderrahim E (2022). Gitelman syndrome, hypomagnesemia, and venous thrombosis: an intriguing association. Clin Case Rep.

[7] Mendes Filho O, Maués CA, De Macedo FP (2020). Neuroendocrine pancreatic tumor causing chronic diarrhea in young adult, a case report. AME Case Rep.

[8] Chan JY, Toh MR, Chong ST (2020). Multiple neoplasia in a patient with Gitelman syndrome harboring germline monoallelic MUTYH mutation. NPJ Genom Med.

[9] Nilubol N, Freedman EM, Quezado MM, Patel D, Kebebew E (2016). Pancreatic neuroendocrine tumor secreting vasoactive intestinal peptide and dopamine with pulmonary emboli: a case report. J Clin Endocrinol Metab.

[10] Fujimura J, Nozu K, Yamamura T (2019). Clinical and genetic characteristics in patients with Gitelman syndrome. Kidney Int Rep.

